# Evaluation of Surfactants on Graphene Dispersion and Thermal Performance for Heat Dissipation Coating

**DOI:** 10.3390/polym14050952

**Published:** 2022-02-27

**Authors:** Chia Cheng, Wen-Hao Shi, Tun-Ping Teng, Chii-Rong Yang

**Affiliations:** 1 Department of Mechatronic Engineering, National Taiwan Normal University, No. 162, Sec. 1, He-ping E. Road, Da-an District, Taipei City 10610, Taiwan; giles540624@gmail.com (C.C.); 40273032h@gmail.com (W.-H.S.); 2 Undergraduate Program of Vehicle and Energy Engineering, National Taiwan Normal University, No. 162, Sec. 1, He-ping E. Road, Da-an District, Taipei City 10610, Taiwan

**Keywords:** water-based epoxy, volatile organic compounds, heat dissipation coating, graphene flakes, surfactant, synergistic effect, infrared emissivity

## Abstract

With the development of thin and high-power electronic devices, heat dissipation has become an important and urgent issue in thermal management. In this study, a water-based epoxy was used as a polymer matrix to prepare heat dissipation coatings utilizing low volatile organic compounds, which were environmentally friendly and had a high heat-dissipating performance. Graphene flakes, multi-walled carbon nanotubes and aluminum oxide particles were used as fillers for preparing the heat dissipation coating. The graphene flakes and multi-walled carbon nanotubes were dispersed in a water-based epoxy by adding sodium dihexyl sulfosuccinate and poly (dimethyldiallylammonium chloride). These two surfactants were combined as a dispersant to improve the dispersibility of the carbon nanomaterials in the water-based epoxy. The synergistic effect of the well-dispersed fillers improved the heat-dissipating performance. The experimental results show that the infrared emissivity of the heat dissipation film was 0.96 after filling 30 wt% aluminum oxide particles, 2 wt% graphene flakes and 2 wt% multi-walled carbon nanotubes into a water-based epoxy. The heat dissipation film reduced the thermal equilibrium temperature of the bare copper panel by 17.8 °C under a heating power of 10 W. The film was applied in a heat dissipation test on a 15 W LED bulb, and the thermal equilibrium temperature was reduced by 21.3 °C. The results demonstrate that the carbon nanomaterial-based heat dissipation coating with a water-based epoxy could significantly reduce the thermal equilibrium temperature, giving a high potential for the application of thermal management.

## 1. Introduction

With the tendency to develop electronic devices with high power, compact size and densified parts, thermal management has become important in the electronic and photoelectric industries [1,2,3,4]. Traditional heat dissipation methods can be divided into two modes: (a) Passive heat dissipation using heat sinks for thermal conversion, and (b) active heat dissipation using fans or water-cooling systems. However, under limited space, size and environment, active heat dissipation cannot be used to exchange heat, and the use of metal fins to radiate heat then becomes the main heat dissipation method [5,6,7,8,9,10,11]. Copper and aluminum have excellent heat conductivity and are often used as heat sink materials for heat dissipation, but they cannot achieve good convective heat transfer under the condition of passive heat dissipation. Therefore, increasing the emissivity of the heat sink metal surface to enhance the radiant heat transfer dissipation efficiency has become an urgent problem. While surface treatment technologies for metal parts, such as electroplating [12,13] and anodizing [14,15], can improve the surface emissivity, they also increase the thermal resistance of the interface. Improving the heat radiation efficiency of metal surfaces through heat dissipation coating techniques is thus an important approach to promote the heat-dissipating performance of metal heat sinks [16,17,18,19,20,21].

Due to their superior heat transfer performance, carbon materials were the focus of earlier studies. Graphite, carbon black, activated carbon and diamond powder have all been used as thermally conductive fillers to enhance the performance of heat dissipation coatings. In 2008, Chen et al. also used a diamond film with a thickness of 20 μm on a silicon wafer as the heat dissipation layer of a gallium nitride (GaN) micro-light-emitting diode (LED). The GaN LED bulb with a diamond heat-dissipating layer could reach a temperature reduction of 20 °C under a working current of 1 A [22]. In recent years, carbon nanotubes and graphene have attracted much attention in the field of heat dissipation applications. In 2009, Chetan et al. developed a molecular fan coating with high surface emissivity, which was used as a thin film for radiative cooling. Nanodiamond powders (NPDs), multi-walled carbon nanotubes (MWCNTs) and carbon black were dispersed in an acrylate emulsion to form composite materials. A fluorosurfactant (Zonyl FS-510) and a poly(tetraflouroethylene) (PTFE) dispersion were added to improve the dispersibility of the carbon material in the solution. They also measured the characteristic peaks of MWCNTs, carbon black and NDPs by Raman spectroscopy, and found that the MWCNTs had the strongest G-band intensity. In other words, MWCNTs had the highest degree of lattice quantization, thus having the highest phonon quantization. Molecular fan coatings with the same 0.7 wt% of NDPs, carbon black and MWCNTs were tested using a 30 W heater, and these NDPs, carbon black and MWCNT coatings could reduce the equilibrium temperature by 9 °C, 11 °C and 13 °C, respectively. Their findings demonstrate that the higher the lattice quantization is, the better the heat-dissipating performance is [2].

In 2013, Hsiao et al. used molecular fans for radiative cooling. Graphene flakes with a high lattice G band and 2D band in the Raman spectra were added into acrylate as coatings to enhance their surface emissivity and heat-dissipating efficiency, and used to evaluate the heat-dissipating characteristics of an LED heat sink. The molecular fan could reduce the equilibrium temperature of a 50 W LED by 16 °C while functioning as a dielectric layer with a high breakdown voltage greater than 5 kV. This coating also could enhance the heat-dissipating efficiency by 20% under constant brightness [23]. In subsequent experiments, the same team filled MWCNTs into acrylic copolymer emulsions, and applied them to an LED heat sink. The molecular fans caused the equilibrium temperature to cool down by 10 °C and 20 °C on the same heat sink at input operating voltages of 35 V and 55 V, respectively [24,25,26]. In 2019, Hong et al. used a spin-assisted layer-by-layer deposition method to alternately stack alumina (Al_2_O_3_) and reduced graphene oxide (rGO) layers for improving the heat conduction path, and applied this film to 60 W LEDs to evaluate their cooling efficiency. The results show that the equilibrium temperature of only LED was 95.3 °C, while the LED coated with pure rGO film was 81.2 °C, showing a temperature difference of 14.1 °C. The equilibrium temperature of the rGO/Al_2_O_3_ composite film was 71.2 °C, reaching a temperature difference of 24.1 °C. This proves that the addition of Al_2_O_3_ powders to the film could increase the heat conduction path and improve the efficiency of the heat-dissipating film [27]. In fact, some references have demonstrated that the different types of filler combinations could enhance thermal conductivity of polymer-based composites, which was attributed to the synergistic effect [28,29,30]. In particular, with the hybrid filler combining 1D carbon nanotube, 2D graphene flake and particle-shaped filler with different sizes, they could maximize the number of thermal conductive pathways and decrease the thermal boundary resistance in polymer composites, and could thus provide faster and more effective pathways for phonon transport.

This study aims to develop a high-performance, high-stability, high-volume production and environmentally friendly carbon nanomaterial-based heat dissipation coating to prepare a heat dissipation film. The main research features include the use of the hybrid filler with good heat transfer characteristics, different sizes and morphologies such as Al_2_O_3_ powders in diameters of 300 nm, 5 μm and 10 μm, 2D graphene flakes (GNFs) and 1D MWCNTs to mix with a water-based epoxy, and use of the synergistic effect among the fillers and the high emissivity of the carbon-based materials produces high-performance heat dissipation coating. The tubular MWCNTs acted as the spacer to prevent 2D graphene flakes from restacking for improving heat-dissipating performance. Secondly, GNFs were prepared by the CO_2_ supercritical exfoliation method, and the preparation process did not use the oxidizing agents or strong acids to meet the trend of environmental protection requirements. Furthermore, the anionic sodium dihexyl sulfosuccinate (SDSS) and cationic poly(dimethyldiallylammonium chloride) (PDDA) as dispersants were used to improve the dispersibility of the additive material in the water-based epoxy and can dilute the heat dissipation coating to a suitable viscosity for the coating process. Materials and finished products at different research stages were tested such as SEM, TEM, AFM, FTIR, Raman spectroscopy and TGA to understand the changes in the morphology and characteristics of the materials. Finally, the heat dissipation coating was coated on the copper substrate to prepare a heat dissipation film to actually test the heat dissipation performance of a 15 W LED bulb to verify the practicability of the heat dissipation coating developed in this research.

## 2. Related Theories

To improve the heat-dissipating performance of a heat sink, heat dissipation coatings composed of GNFs, MWCNTs, Al_2_O_3_ particles and a water-based epoxy were used to achieve a synergistic effect. The thermal radiation energy (*Q_r_*) (W/m^2^) dissipated from the surface of the material could be estimated by the Stefan–Boltzmann equation, shown as equation (1):

(1) $$Q_{r}=\varepsilon \cdot A\cdot \sigma \cdot T^{4}$$

where *ε* is the emissivity; *A* is the surface area (m^2^); *σ* is the Boltzmann constant (5.6697 × 10^−8^ W/m^2^ K^4^); and *T* is the surface temperature (K) [31,32]. Improved emissivity could increase the heat radiation energy dissipated on the surface of the material and improve the heat-dissipating efficiency of the metal fins. Enhancing the heat radiation emissivity of the heat dissipation coating is an effective method to increase the *Q_r_* value of metal fins with a fixed surface area. However, there is no perfect black body material (ε = 1) in nature; only the emissivity of graphene and carbon nanotubes are close to black body materials (ε = 0.99), and they are often used as filling materials for heat dissipation coatings [33]. In this study, the GNFs were prepared using a CO_2_ supercritical exfoliation method, and were added along with MWCNTs into the coating as fillers. Tubular MWCNTs were used to prevent the restacking of the GNFs. These carbon nanomaterials increased the emissivity of the heat dissipation coating, and enhanced their heat-dissipating capacity to provide a good cooling effect.

Graphene is a single-atom-thick, two-dimensional material with a hexagonal honeycomb crystal lattice, in which carbon atoms consist of sp^2^ hybrid orbitals. Because of the large Van der Waals force, GNFs can easily agglomerate and negatively affect the performance of a product. Therefore, adding a dispersant to graphene-based coatings is necessary to increase the even distribution of GNFs in the coatings. The principle of graphene dispersion is to make the force between the solvent molecules be the same as the force between the graphite layers. Surfactants are usually added to adjust the surface tension of the solution to make it close to the surface energy of the graphene. This theory is expressed in Equation (2) [34,35,36]:

(2) $$\frac{H_{\mathrm{mix}}}{V_{\mathrm{mix}}}=\frac{2}{T_{G}}\;{\left(\sqrt{E_{\mathrm{SS}}}-\sqrt{E_{\mathrm{SG}}}\right)}^{2}{\;\Phi }_{G}$$

where H_mix_ is the enthalpy of mixing; V_mix_ is the volume of the solution; T_G_ is the thickness of the GNFs (nm); E_SS_ and E_SG_ are the surface tension of the solution and the surface energy of the graphene, respectively; Φ_G_ is the volume fraction of the dispersant; H_mix_ is the energy required for graphene to be dispersed or exfoliated. According to equation (2), when E_SS_ and E_SG_ are closer to each other, H_mix_ is closer to 0. When H_mix_ is the smallest, the energy needed to exfoliate graphene is relatively small. With a smaller H_mix_, less energy is required to disperse or exfoliate the graphene, which in turn leads to better dispersion effect of the graphene [37,38,39]. The surface energy of GNFs is 45 mJ/m^2^, and the surface tensions of N-methylpyrrolidone and dimethylformamide are 40.1 mJ/m^2^ and 37.1 mJ/m^2^, respectively. Many studies added these two organic solvents to achieve the effect of dispersing GNFs, but these solvent-type dispersants are expensive and toxic, so they are not suitable in terms of environmental friendliness and health. On the other hand, the formulation of aqueous graphene suspensions has received increasing attention. The surface tension of water is 71−72 mJ/m^2^, and aqueous graphene suspensions generally use sodium dodecyl benzene sulfonate (SDBS) as a dispersant [40]. The surface tension of the suspension is 37−41 mJ/m^2^ depending on the SDBS concentration, which can help GNFs to disperse in aqueous solutions [41,42]. Recent literature has proposed the research results on ionic-typed dispersants, which are inexpensive, non-toxic and easy to manufacture. Ionic-typed surfactants can be added to water so that the surface tension of the solution is reduced from 71−72 mJ/m^2^ to 40 mJ/m^2^, which can be used to improve the dispersion of GNFs in aqueous solutions. This study used two ionic surfactants to formulate a novel dispersant that could adjust the surface tension of aqueous solutions, thereby providing a suitable dispersion range for GNFs and MWCNTs (36.7–46.5 mJ/m^2^) [43,44]. The dispersant could evenly disperse the carbon nanomaterials in a water-based epoxy resin and improve the performance of the heat dissipation film.

**Figure 1:**
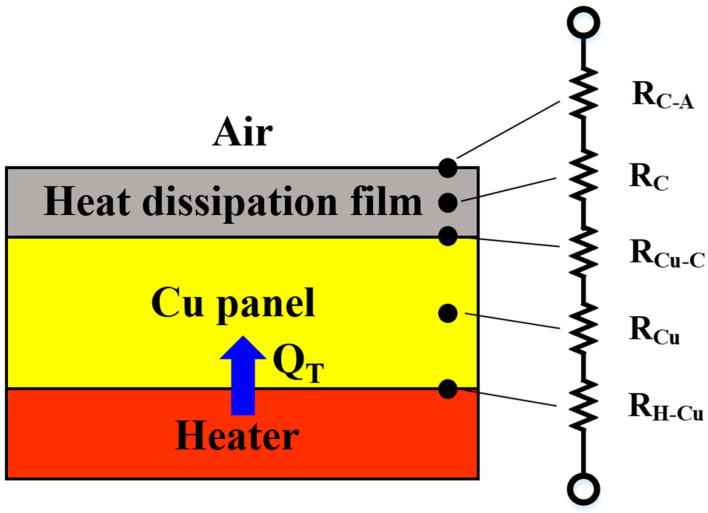
Schematic of the R_total_ from the heater to the outside ambience through the bare copper panel and heat dissipation film**.**

This study analyzed the total thermal resistance (R_total_) of the heat flow (Q_T_) from the heater to the air, which passes through the copper heat sink, the heat dissipation film and the interface of these materials, as shown in Figure 1. The thermal resistance is expressed by Equation (3):

R_total_ = R_H-Cu_ + R_Cu_ + R_Cu-C_ + R_C_ + R_C-A_ (3)

where R_total_ is the total thermal resistance of the heat transfer; R_H-Cu_ is the thermal resistance of the interface between the heater and the copper heat sink; R_Cu_ is the thermal resistance inside the copper heat sink; R_Cu-C_ is the thermal resistance of the interface between the copper heat sink and the heat dissipation film; R_C_ is the thermal resistance of the heat dissipation film; and R_C-A_ is the thermal resistance of the interface between the heat dissipation film and the air. In this study, Al_2_O_3_ and carbon nanomaterials were added to the film to induce a synergistic effect that could improve the thermal conductivity of the film and reduce the R_C_ value, while increasing the emissivity of the heat dissipation film to reduce the R_C-A_. Furthermore, the excellent adhesion between the heat dissipation film and the copper heat sink also reduced the R_Cu-C_. By reducing the R_C_, R_C-A_ and R_Cu-C_, the overall thermal resistance (R_total_) decreased, and the heat energy was more easily transferred from the heater to the air, thereby improving the heat-dissipating performance. 

## 3. Experimental Methods and Procedures

### 3.1. Formulation of the Carbon Nanomaterial Dispersant

In this study, two ionic surfactants were used to formulate dispersants for dispersing the carbon nanomaterials; one was a cationic PDDA (DKS Co., Ltd., Kyoto, Japan) for dispersibility, and the other was an anionic SDSS (Cytec Industries Inc., Woodland Park, NJ, USA) for wettability. The addition ratio of these two surfactants was adjusted to make the surface tension of the dispersant reach 36.7–46.5 mJ/m^2^, which could make the carbon nanomaterials achieve the best dispersion effect in the aqueous solution. The addition of PDDA could stably disperse the carbon nanomaterials in the solution, while the addition of SDSS could increase the wettability of the carbon nanomaterials in the water-based epoxy. Thus, the thermal resistance in the interface between the carbon nanomaterials and the epoxy matrix could be reduced in the coating. In fact, PDDA and SDSS also performed the role of a dispersion and intercalation compound in the CO_2_ supercritical exfoliating graphene process, as described in Section 3.2. In this study, the dispersant of carbon nanomaterials was formulated by adding a specific proportion of 0.05 wt% SDSS and 1 wt% PDDA to deionized water, which was then stirred for 12 h using a magnetic stirrer. The surface tension of this dispersant was measured by a surface tension meter (First ten Ǻngstroms, FTA-125, Newark, NJ, USA). Suspensions made of 0.5 wt% carbon nanomaterials in deionized water with dispersants were prepared by sonication for 10 min, and then stood for three days. Finally, the sedimentation method was used to judge the degree of dispersion of the carbon nanomaterials.

### 3.2. Preparation of Graphene flakXes by Supercritical CO_2_ Assisted Exfoliation

Some recent studies have proposed CO_2_ supercritical fluid methods for preparing GNFs, but they have some disadvantages, including the low mass production of few-layer GNFs, the use of expanded graphite as a raw material (which requires several supercritical fluid circulation treatments) and the need to use organic solvents (N-methylpyrrolidone, N,N-dimethylformamide) as supercritical fluids [45,46]. In addition, some studies used surfactants (sodium dodecyl sulphate (SDS) and sodium dodecylbenzene sulfonate (SDBS)) to assist the graphene exfoliation process [47,48]. Herein, surfactants were added to avoid the interaction of Van der Waals forces to restack the GNFs. When supercritical CO_2_ was passed into the vessel containing the graphite solution with a surfactant, the graphite layers were intercalated with the CO_2_ supercritical fluid. As the supercritical CO_2_ returned to a gaseous state upon rapid depressurization of the vessel, the CO_2_-intercalated graphite was forced to exfoliate or delaminate by the expansion of the supercritical CO_2_ disposed interstitially between the layers [48]. To increase the yield rate of the GNFs, Gao et al. also added an ultrasonic oscillation procedure after supercritical CO_2_ assisted exfoliation processing to improve the peeling effect of intercalated graphite [49].

**Figure 2:**
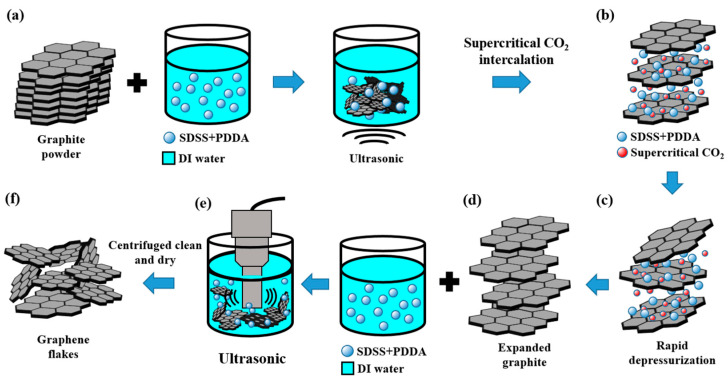
(**a**–**f**) Flow chart of preparing the GNFs using the supercritical CO_2_ and surfactant treatment. (**a**) Prepare graphite solution and ultrasonically agitated. (**b**) Supercritical CO_2_ intercalation. (**c**) Rapid depressurization. (**d**,**e**) Prepare expanded graphite solution and horn sonication. (**f**) Centrifuged clean and dry to collect graphene flakes.

This study developed an environmentally friendly and energy-saving process by using a pure aqueous solution of graphite and adding a special surfactant to increase the wettability and dispersibility of the graphite. A CO_2_ supercritical fluid was further used to enhance the intercalation and exfoliation of the graphite. Under the synergistic effect of the CO_2_ supercritical fluid and the surfactant, the effect of increasing the layer spacing or exfoliating the graphite layer was achieved, and high-quality and high-yield GNFs were prepared with the aid of subsequent ultrasonic oscillations. Figure 2 shows the flow chart of preparing GNFs using the supercritical CO_2_ fluid and surfactant treatment. The steps are as follows. First, a graphite solution with 0.05 wt% SDSS, 1 wt% PDDA surfactants, and 10 g of graphite powder (200 mesh, Showa Kako, Japan) was prepared and ultrasonically agitated for 10 min to increase the wettability of the graphite powder, as shown in Figure 2a. Then, the graphite solution was put into the CO_2_ supercritical machine in which CO_2_ gas turns to a supercritical state under the conditions of 50 °C and 2000 psi, as shown in Figure 2b. Due to the low viscosity and high diffusion coefficient of the supercritical fluid, the CO_2_ supercritical fluid along with the PDDA and SDSS molecules and the water molecules were intercalated between the graphite layers. The supercritical fluid had almost no surface tension, so it easily penetrated into the graphite layer. After the graphite aqueous solution was treated for 2 h using the CO_2_ supercritical fluid, the pressure was quickly released, as shown in Figure 2c. The volume expansion pressure generated by the vaporization of the CO_2_ fluid between the graphite layers instantly exfoliated the graphite layer by layer. To increase the yield rate of the GNFs, the intercalated and expanded graphite was added into the 0.05 wt% SDSS and 1 wt% PDDA solution, then further horn sonication (Q700, Misonix Qsonica, Newtown, CT, USA) was conducted for 30 min, as shown in Figure 2d,e. After the ultrasonic oscillation, the GNF solution was centrifuged at 8500 rpm for 90 min to separate the GNFs. The GNF slurry was further dissolved repeatedly three times in 95% ethanol and recovered after centrifugation at 8500 rpm for 90 min to remove the unwanted PDDA and SDSS molecules. Finally, the GNF slurry was collected and dried in the shade, as shown in Figure 2f. This method was a process of mechanically exfoliating graphene flakes, which did not destroy the surface morphology of the graphene flakes or allow them to be grafted with other functional groups. These GNFs could maintain good heat transfer characteristics to enhance the heat-dissipating performance of the heat dissipation coating. Figure S1 shows the equipment diagram of the CO_2_ supercritical machine. A pump and a heater were used to provide the pressure and temperature, and the heater was equipped with a temperature controller so that the CO_2_ could reach the supercritical state and the graphite powders could be fully mixed with the supercritical CO_2_ fluid in the reaction tank. Finally, the pressure was quickly relieved to exfoliate the graphite and produce the GNFs used in this study.

### 3.3. Formulation of the Heat Dissipation Coating

**Figure 3:**
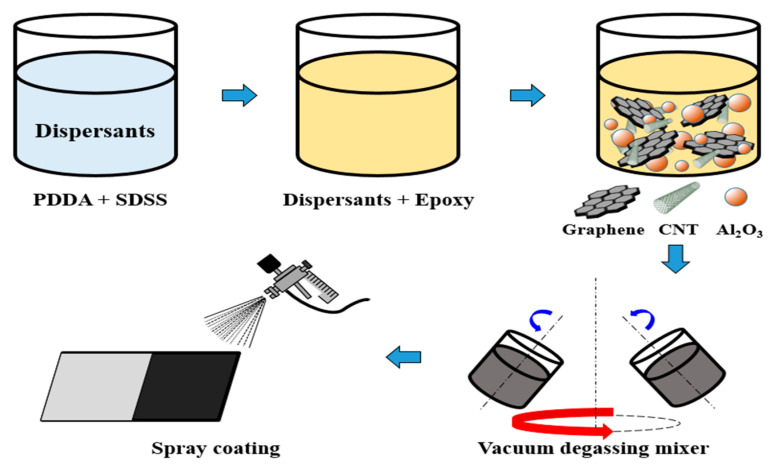
Preparation process of the graphene heat dissipation coating.

In this study, a water-based epoxy (902AB, Perma Enterprise Co., Ltd., Taoyuan City, Taiwan) was used as the polymer matrix of the coating. Spherical Al_2_O_3_ particles with different diameters of 5 μm and 10 μm (Inno-trust Tech Co., Ltd., Taichung City, Taiwan) and irregularly shaped Al_2_O_3_ particles of 300 nm (Jzuh Chia International Corp., Taipei, Taiwan) were used as the filler particles. The mixed different-sized particles made the Al_2_O_3_ inside the coating more compact, thus increasing the heat conduction path and improving the heat conduction performance [50]. In addition, the GNFs prepared by the CO_2_ supercritical exfoliation and MWCNTs (99% pure, OD = 10−20 nm, Golden Innovation Business Co., Ltd., New Taipei City, Taiwan) were used as the filled carbon nanomaterials. The use of tubular MWCNTs could prevent 2D graphene flakes from restacking to improve heat-dissipating performance. Figure 3 shows the blending process of the heat dissipation coating. The dispersant prepared in Section 3.1 was used to dilute the epoxy to a viscosity of about 1500 cps for suitably adding the fillers. The 300 nm-sized, 5 μm-sized and 10 μm-sized Al_2_O_3_ particles were filled into the diluted epoxy at a ratio of 1:1:1 to increase the thermal conductivity of the coating. Moreover, an appropriate number of GNFs and MWCNTs were added to increase the thermal conductivity and emissivity of the coating in order to achieve a good cooling effect. At the same time, the synergistic effect of the carbon nanomaterials and Al_2_O_3_ particles increased the thermal conductivity and heat-dissipating efficiency. A planetary vacuum degassing mixer (MV-300S, Chau Guan Technology, New Taipei City, Taiwan) was used to fully mix the water-based epoxy and fillers to complete the preparation of the heat dissipation coating. The heat dissipation coating was sprayed on the copper heat sink for the cooling tests. In this study, the viscosity of the coating was measured by a digital viscometer (Brookfield, DV-I Prime, Middleboro, MA, USA), and the viscosity of the coating was controlled to within 1500 cps. The viscosity of the heat dissipation coating was controlled by the ratio of Al_2_O_3_ particles to carbon nanomaterials for fluidity. It was expected that a carbon nanomaterial-based heat dissipation film with a thickness of about 30 μm could be obtained for good heat-dissipating performance.

### 3.4. Evaluation of the Emissivity and Heat-Dissipating Performance

**Figure 4:**
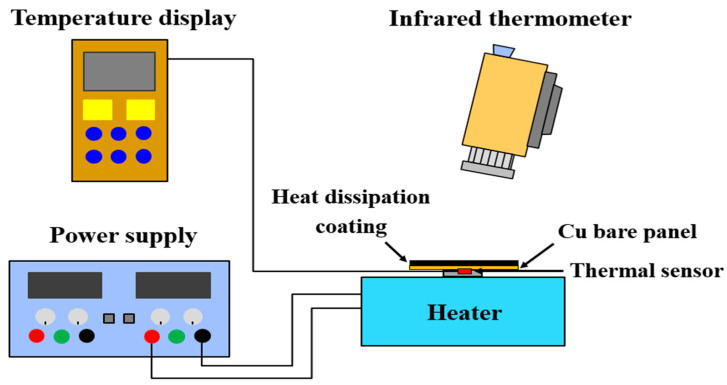
Schematic of the temperature monitoring system and thermal emissivity measurement.

The cooling efficiency of the heat dissipation coating was evaluated by a temperature-monitoring system (Figure 4). This system consists of four parts: A power supply, a heater (0−60 W), a thermocouple and a temperature monitor. An IR thermal imaging camera (AVIO, TVS-200, Yokohama, Japan) was used to measure the thermal emissivity of the heat dissipation coatings. The power of the heater was adjusted by a power supply. The heat source was fixed at a 10 W output, which was controlled by a variable transformer. The temperature of the heat dissipation film was monitored by a thermocouple and was recorded every 30 s. The temperature of the panel was equilibrated with the surroundings after heating for 60 min. The copper heat sink was in the size of 5 cm × 5 cm × 1 mm, and was cleaned with alcohol and acetone to remove surface dirt and grease. The coating was sprayed on the panel by a spray gun, followed by drying at 80 °C for 30 min, resulting in a heat dissipation film on the panel with a thickness of about 30 μm, as measured by a thickness gauge. The thermal emissivity was measured by recording the actual temperature of the heat sink by the thermal couple. Then, the emissivity of the infrared thermal imaging camera was adjusted so that the measured temperature could be the same as the actual temperature of the heat sink. At this time, the emissivity displayed on the infrared thermal imaging camera represented the emissivity of the heat dissipation film.

## 4. Results and Discussion

### 4.1. Morphology Measurement of the Supercritical CO_2_ Exfoliated GNFs

**Figure 5:**
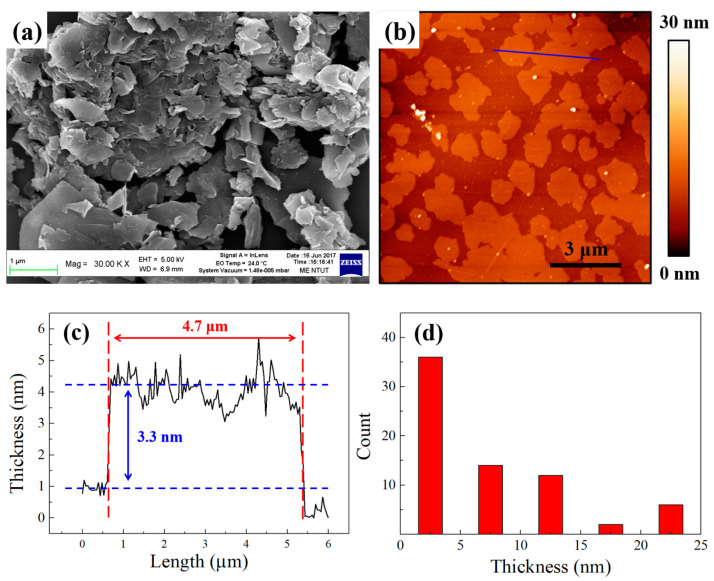

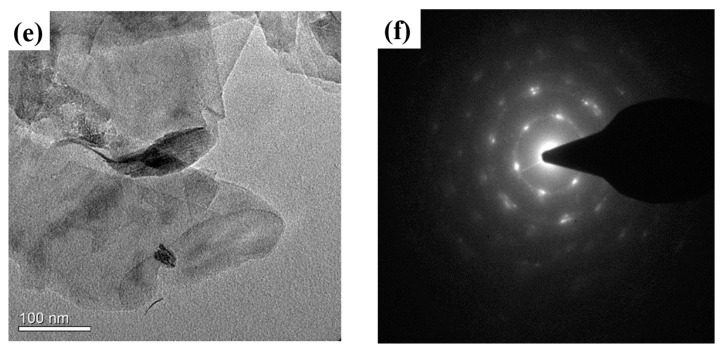
(**a**) SEM image of the GNFs; (**b**) AFM image of the GNFs; (**c**) thickness profile of the GNFs; (**d**) thickness distribution map of the GNFs; (**e**,**f**) TEM image of the GNFs and their SAED pattern.

An atomic force microscope (AFM) (FSM Nanoview1000, Utek Material Co., Ltd., Taipei City,Taiwan) and scanning electron microscope (SEM) (JSM-7610F, JEOL, Tokyo, Japan) were used to measure the surface morphology of the exfoliated GNFs, as shown in Figure 5. Figure 5a shows the SEM image of the GNFs, which expresses a sheet-like morphology. It was predicted that these GNFs have good thermal conductivity and that applying them to the heat dissipation coating of this study could better improve the heat-dissipating effect. In addition, the AFM images shown in Figure 5b,c indicate that the thickness of the GNFs was about 3–5 nm, therefore they could be regarded as few-layer GNFs [48]. The average lateral size of the GNFs was about 5 μm. A larger sheet diameter in the GNFs could effectively increase the heat transfer path, and reduce the thermal resistance effect generated when the GNFs with smaller sheet diameters overlapped each other, so they had higher thermal conductivity. Figure 5d shows the thickness distribution of the GNFs after the CO_2_ supercritical assisted exfoliation process was used. As seen, nearly 85% of the GNFs had a thickness below 15 nm. This suggests that the prepared GNFs have a larger sheet diameter, complete sheet morphology, and a few-layer thickness distribution due to the CO_2_ supercritical assisted exfoliation method. These GNFs were added into the heat dissipation coating not only to increase the emissivity, but also to improve the heat-dissipating effect with their good thermal conductivity. A transmission electron microscope (TEM) (JEM-2100F, JEOL, Tokyo, Japan) was used to verify the very thin thickness and good lattice characteristics of the exfoliated GNFs. The TEM image of the GNFs and their selected area electron diffraction (SAED) in Figure 5e,f show that the CO_2_ supercritical assisted process could exfoliate the graphite into few-layer and high-quality GNFs. The SAED graph shows an obvious hexagonal lattice structure, which also proves that the exfoliated GNFs still retain excellent crystalline characteristics.

### 4.2. Effect of Surfactants on the Dispersibility of Carbon Nanomaterials

**Figure 6:**
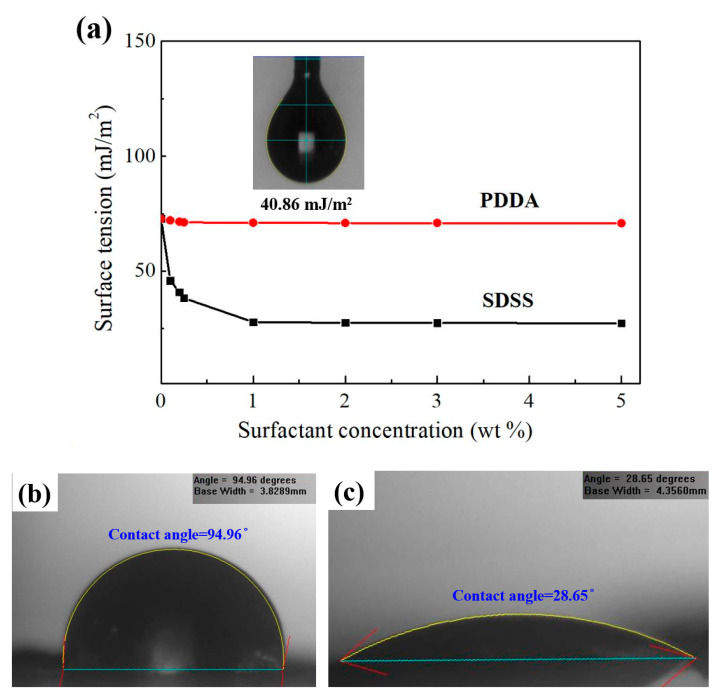
(**a**) Effect of surfactant concentrations on the surface tension of deionized water. The inset shows that a surface tension of 40.86 mJ/m^2^ was obtained from the pendant drop shape analysis when the addition ratio was 1 wt% PDDA + 0.05 wt% SDSS. Contact angle measurement (**b**) with a deionized water droplet, (**c**) with a dispersant droplet on the carbon nanomaterial film.

The relationship between the concentration of the surfactant and the surface tension of the solution was discussed to formulate dispersants for dispersing carbon nanomaterials. Figure 6a shows the surface tension caused by different concentrations of anionic PDDA and cationic SDSS surfactants when dissolved in deionized water. The figure shows that the surface tension of the SDSS at a concentration of 0.5 wt% could be quickly reduced from 72 mJ/m^2^ to below 40 mJ/m^2^. When the concentration of the SDSS was above 1 wt%, the surface tension was 27 mJ/m^2^. In contrast, when adding the PDDA surfactant, the surface tension could only be reduced to 70 mJ/m^2^. Regardless of the increase of the concentration of PDDA, the surface tension could not be reduced to 40 mJ/m^2^, showing the difference between the SDSS and PDDA surfactants. Although the PDDA could not effectively reduce the surface tension of the solution, it still had the effect of dispersing materials, which was verified by later experiments. In order to formulate a solution with a surface tension of 40 mJ/m^2^ (which was most suitable for dispersing the carbon nanomaterials), this study formulated 1 wt% PDDA + 0.05 wt% SDSS as the best addition ratio of dispersants, and the surface tension of the solution was 40.86 mJ/m^2^. The inset in Figure 6a shows the drop shape of the dispersant pendant obtained at this addition ratio. The surface tension was measured in the pendant drop shape analysis. This dispersant, with a surface tension of 40.86 mJ/m^2^, was used to prepare the carbon nanomaterial suspensions. Moreover, we also measured the contact angle to reflect the activation energy required for movement of a water droplet on the surface of graphene flakes, and evaluated the wettability effect of dispersants. Figure 6b shows the contact angle of 94.96° measured with a deionized water droplet on the carbon nanomaterial film; Figure 6c shows the contact angle of 28.65° measured with a dispersant droplet on the carbon nanomaterial. It is confirmed that the affinity between dispersants and carbon nanomaterials is obviously greater than deionized water only, so carbon nanomaterials can be dispersed better in the aqueous solution with a dispersant.

A number of solutions containing different surfactants were formulated and applied to the carbon nanomaterial suspensions for a sedimentation comparison, including 0.2 wt% SDSS (40.8 mJ/m^2^), 1 wt% PDDA (71 mJ/m^2^), 1 wt% PDDA + 0.05 wt% SDSS (40.86 mJ/m^2^) and 1 wt% SDBS (41 mJ/m^2^) solutions. The concentration of the carbon nanomaterial suspensions was 5 mg/mL, which underwent ultrasound oscillation for 10 min. A carbon nanomaterial suspension was also formulated using pure deionized water (72 mJ/m^2^) and used as a reference for comparison. The suspensions were allowed to stand for three days to observe the precipitation phenomenon, and the effects of different dispersants on the GNF suspensions and MWCNT suspensions are shown in Figures S2 and S3, respectively. Figure S2 shows that when the standing time reached 1 h, the GNFs in the pure water had already produced some preliminary precipitation. The GNF suspensions with the additions of SDSS, PDDA, SDSS+PDDA and SDBS still maintained a good dispersion effect. However, when the standing time reached three days, the suspension of pure SDSS and pure PDDA showed partial precipitation, the suspension of SDBS showed full precipitation, and the suspension of SDSS+PDDA maintained a good dispersion effect. It was, therefore, proved that the dispersion effect of SDSS+PDDA was better than SDBS, which is generally used as the dispersant in the aqueous suspension of GNFs. In addition, PDDA also had the obvious effect of increasing the dispersibility of the GNFs. Therefore, while the surface tensions of SDSS and SDSS+PDDA were almost the same, the solution of SDSS+PDDA still had the best dispersion effect. The sedimentation experiment of the MWCNT suspensions followed the same procedure as the dispersibility test of the GNF suspensions. Figure S3 shows that when the standing time reached 10 min, the MWCNTs in the pure water had already produced some obvious precipitation. The MWCNT suspensions with the addition of PDDA, SDSS+PDDA, and SDBS still maintained a good dispersion effect. However, when the standing time reached three days, for the suspensions in deionized water, SDSS showed precipitation, and the suspension of PDDA, SDSS+PDDA and SDBS still maintained a good dispersion effect. While the dispersion effect of the MWCNTs in the SDSS+PDDA and SDBS was very good, the dispersion of the GNFs in the SDSS+PDDA was better than that in the SDBS. Therefore, this study finally selected SDSS+PDDA as the dispersant for the carbon nanomaterials. This dispersant was suitable for diluting the water-based epoxy and adjusting the viscosity of the coating for process requirements. Furthermore, it could improve the dispersion effect of carbon nanomaterials in the coating by reducing the thermal resistance in the interface between the carbon nanomaterials and epoxy matrix in the coating, thereby enhancing the heat-dissipating effect of the heat dissipation coating.

**Figure 7:**
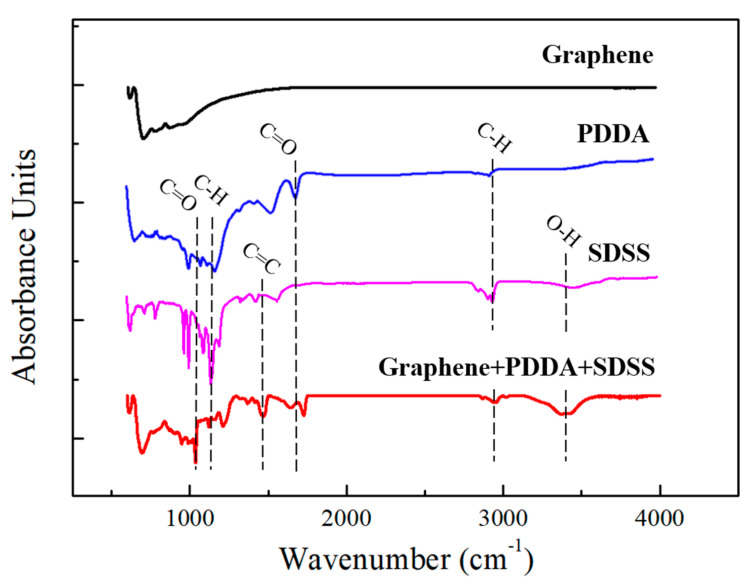
FTIR spectra of the intrinsic GNFs and GNFs treated with SDSS and PDDA surfactants.

In order to explore the influence of the functional groups of SDSS and PDDA on the dispersibility of the GNFs, it was necessary to investigate why PDDA was not helpful in decreasing surface tension, but helpful to the dispersibility of GNFs. This study then measured the functional groups on the surface of the GNFs formulated with deionized water, SDSS, PDDA, and SDSS+PDDA by a Fourier-transform infrared spectrometer (FTIR) (Bruker, Vertex 80v, USA), as shown in Figure 7. As seen, the black curve is the measurement result of the GNFs without surfactants, while the blue curve and the pink curve are the measurement results of only using PDDA and SDSS, respectively. The red curve is the measurement result of the GNFs with the SDSS+PDDA surfactant. Figure 7 shows that many functional groups were grafted on the surface of the GNFs through the modification of the dispersant. The characteristic peaks are shown from the SDSS+PDDA curve, in which 1162 cm^−1^ and 1726 cm^−1^ represent the C=O bonding, 1210 cm^−1^ and 2964 cm^−1^ represent the CH bonding, 1468 cm^−1^ represents the C=C bonding and 3369 cm^−1^ represents the O-H bonding. Among them, the functional groups of 1468 cm^−1^ and 1726 cm^−1^ were provided by PDDA, while the functional groups of 1162 cm^−1^ and 3369 cm^−1^ were provided by SDSS. Through these grafted functional groups, the GNFs could be more uniformly dispersed in the solution. Therefore, this study used the SDSS+PDDA surfactant as the dispersant. The wettability of the SDSS promoted the adhesion of the carbon nanomaterials and water-based epoxy to reduce the interface thermal resistance of the interface. The dispersibility of the PDDA prevented the GNFs from agglomerating in the heat dissipation coatings. The synergistic effect of these two surfactants increased the thermal conductivity of the coating and the heat transfer path so that the performance of the heat dissipation coating could be improved.

### 4.3. Characteristic Analysis of the Heat Dissipation Coatings

**Figure 8:**
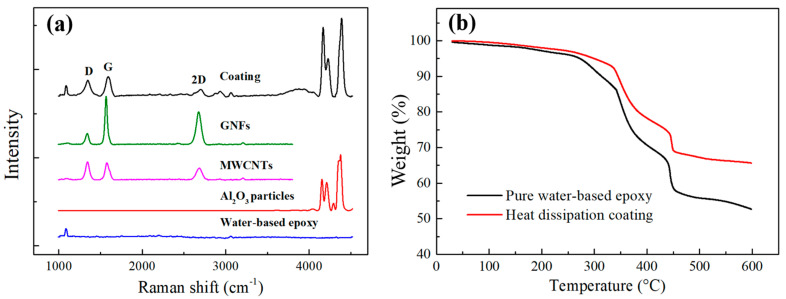
(**a**) Comparison of the Raman spectra for the different materials in coating; (**b**) TGA curves of the pure epoxy and heat dissipation coating.

In order to prove that the GNFs exfoliated with the CO_2_ supercritical assisted method have good lattice characteristics, which will be advantageous concerning promotion of the heat-dissipating effect of the coating, in this study, the epoxy, Al_2_O_3_ particles, GNFs, MWCNTs and heat dissipation coating were measured by a Raman spectrometer (532.15 nm, NRS 4100, Jasco, Japan), as shown in Figure 8a. The blue curve is the result of the pure epoxy. The characteristic peak of the pure epoxy is 1086 cm^−1^. The red curve is the result of the Al_2_O_3_. The characteristic peaks of the Al_2_O_3_ are 4157 cm^−1^, 4213 cm^−1^ and 4380 cm^−1^. The pink curve and the green curve are the measurement results for the MWCNTs and GNFs, respectively. The characteristic peaks are a D-band of ~1350 cm^−1^, a G-band of ~1580 cm^−1^ and a 2D-band of ~2700 cm^−1^. In our measurements, the I_G_/I_D_ of the GNFs is 5.04 and the full width at half maximum (FWHM) of the G-band is 34 cm^−1^. The high G-band peak shows that the carbon nanomaterials have good lattice quantification characteristics, which could improve the heat-dissipating efficiency of the coating. The black curve is the result of the heat dissipation coating. The characteristic peaks of the epoxy, Al_2_O_3_ and carbon nanomaterials were also retained in the heat dissipation coating. The existence of the G-band peak indicates that carbon nanomaterials have good lattice quantification. The influence of the degree of lattice quantization on the emissivity is expressed by Equation (4) [2]:

(4) $$\kappa_{\mathrm{ZZ}}={\sum C\;\upsilon_{Z}}^{2}\tau$$

where *κ*_zz_ is the phonon heat transfer tensor, and C, *υ*_z_ and *τ* are the specific heat, velocity and relaxation time of all phonons, respectively. When these quantized lattice modes are excited by the heat source at a higher temperature, they are promoted to higher quantum states and then decay to the ground state by emitting an infrared photon of energy to dissipate the heat. However, when the degree of lattice quantification is not good, the relaxation time (*τ*) is shorter, resulting in a decrease in the phonon heat transfer tensor (*κ*_zz_), thereby reducing the heat radiation efficiency of the heat dissipation coating and leading to poor heat dissipation [2]. 

In order to verify the thermal stability of the heat dissipation coating to ensure that the coating could be applied to electronic components or LED heat sinks, the thermal stability of the heat dissipation coatings was measured by thermogravimetric analysis (TGA) (Mettler-Toledo, 2-HT, Nänikon, Switzerland), as shown in Figure 8b. The samples were heated to 600 °C at a heating rate of 5 °C per minute. In this study, two types of samples containing pure epoxy and a heat dissipation coating were measured, and the temperature was observed when the weight of the samples reached a weight ratio of 95 wt%. This temperature was defined as the degradation temperature (T_G_). The pure epoxy sample maintained a weight ratio of 95 wt% at 272.42 °C. The sample with the heat dissipation coating maintained a weight ratio of 95 wt% at 299 °C. The higher degradation temperature indicated that the temperature and weight loss of the heat dissipation coating were less than those for the pure epoxy. The carbon nanomaterials and Al_2_O_3_ particles in the heat dissipation coating remained stable at 200 °C. Generally, the working temperature range of the heat dissipation coating ranged from 80 °C to 200 °C. Therefore, the measurement results of the TGA show that the thermal stability of the heat dissipation coatings prepared herein could be applied to general electronic and photoelectric devices.

### 4.4. Characteristic Analysis of the Heat Dissipation Coatings

**Figure 9:**
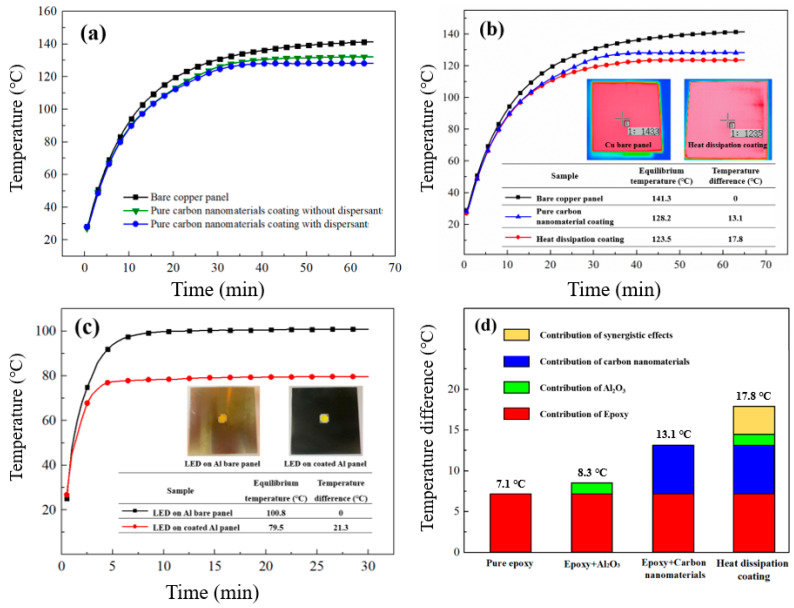
Temperature profile versus operation time curves of: (**a**) A bare copper panel and coating with or without dispersion; (**b**) a bare copper panel and different loadings of graphene coatings; (**c**) the 15 W LED bulb dissipating test. (**d**) Synergistic effect on the enhancement of heat-dissipating performance from various fillers.

Figure 9 shows various coatings that were coated onto a bare copper panel to measure their temperature change, which was then compared to the final equilibrium temperature with or without a heat dissipation coating under a 10 W heater as the heat source. This study first compared the effect of the 2 wt% GNF and 2 wt% MWCNT coatings with or without a 1 wt% PDDA + 0.05 wt% SDSS dispersant for heat-dissipating efficiency. In Figure 9a, the black curve is the heating curve of the bare copper panel, which has a final temperature of 141.3 °C. The red curve is the heat dissipation coating formulated with deionized water only, which has a final temperature of 132.2 °C. The blue curve is the heat dissipation coating formulated with the PDDA+SDSS dispersant, which has a final temperature of 128.2 °C. These results show that the heat dissipation coating formulated with deionized water could only achieve a temperature difference of 9.1°C, thus proving that the heat dissipation coating containing added carbon nanomaterials had a good heat-dissipating effect. In addition, the heat dissipation coating formulated with the PDDA+SDSS dispersant could achieve a temperature difference of 13.1 °C. The added dispersant further reduced the temperature of the heat sink to 4 °C. The PDDA+SDSS dispersant helped the carbon nanomaterials to be uniformly dispersed in the water-based epoxy, giving the heat dissipation coating good heat transfer characteristics and improving the heat-dissipating effect. For confirming the effect of dispersant on the dispersibility of carbon nanomaterials, the energy dispersive spectroscopy (EDS) mapping (JSM-7610F, JEOL, Tokyo, Japan) was also used to identify the distribution of carbon nanomaterials in the coatings, as shown in Figure S4. The EDS mappings in Figure S4a,b were obtained from the heat dissipation coatings added with deionized water only and with dispersant, respectively. It is obvious that Figure S4b has more uniform dispersibility in the carbon element than Figure S4a, which shows the carbon nanomaterials are more uniformly dispersed in the coating with a dispersant. This result also explains why the added dispersant can further reduce the temperature of the heat sink to 4 °C, as shown in Figure 9a. 

Figure 9b shows the temperature versus time curves for different coatings formulated with the PDDA+SDSS dispersant and the uncoated copper panel. The black curve is the heating curve of the bare copper panel and serves as a reference. The blue curve is the heating curve of the heat dissipation coating with 2 wt% GNFs and 2 wt% MWCNTs. The red curve is the heating curve of the copper heat sink coating with 2 wt% GNFs, 2 wt% MWCNTs and 30 wt% Al_2_O_3_ particles. Three types of heat sinks were heated on a 10 W heater for 60 min. The final equilibrium temperature of the bare copper panel was 141.3 °C, while that for the pure carbon nanomaterial heat dissipation coating was 128.2 °C. Compared with the bare copper panel, the temperature difference reached 13.1 °C for the pure carbon nanomaterial heat dissipation coating. Furthermore, the heat dissipation coating using carbon nanomaterials and Al_2_O_3_ particles had the best cooling performance, with a final equilibrium temperature of 123.5 °C. The temperature difference with the bare copper panel reached 17.8 °C. In addition, the result of the cooling test can be observed from the infrared thermal image. The infrared thermal image on the left is the temperature of the bare copper panel (141.3 °C), and the infrared thermal image on the right is the temperature of the heat dissipation coating (123.5 °C). This result demonstrates that the heat dissipation coatings containing GNFs, MWCNTs, and Al_2_O_3_ particles, and the PDDA+SDSS dispersant has the best effect on suppressing temperature rise and promoting the heat-dissipating performance. Finally, the heat dissipation coating with the best performance was applied to a 15 W LED bulb dissipating test, and was coated on the aluminum heat sink of the LED bulb. The equilibrium temperature without or with the heat dissipation coating after lighting for 30 min was compared, as shown in Figure 9c. The final equilibrium temperature of the LED without the heat dissipation coating was 100.8 °C, while that for the LED with the heat dissipation coating was 79.5 °C. This proves that the heat dissipation coating developed herein could make a 15 W LED bulb cool down by 21.3 °C, which meets the expected efficiency, luminance, and product life of the LED bulbs aimed by the industry. Moreover, some experiments were further conducted to demonstrate the synergistic effect of the hybrid filler combination (1D MWCNTs, 2D GNFs and particle-shaped Al_2_O_3_). The use of MWCNTs could prevent GNFs from restacking to improve heat-dissipating performance. Four types of coatings were formulated and their temperature differences were compared using a 10 W heater, as shown in Figure 9d. We found that the temperature difference of pure epoxy, epoxy+Al_2_O_3_, epoxy+carbon nanomaterials and optimal heat dissipation coating was 7.1 °C, 8.3 °C, 13.1 °C and 17.8 °C, respectively. The individual contribution of temperature difference is 1.2 °C and 6 °C from Al_2_O_3_ powder and carbon nanomaterials. However, when these two filler materials were blended together with epoxy, the temperature difference could further improve by 3.5 °C, which was attributed to the synergistic effect of the hybrid filler combination.

This study analyzed the relationship between the heating rate and the heat-dissipating performance of different heat dissipation coatings, as shown in Table S1. When the coating was in the heating stage, a curve with a larger slope indicated a faster heating rate and worse heat dissipation. The various heating curves in Figure 9a–c show that the heating rate stabilized after about 30 min. Therefore, linear regression analysis was used to process the experimental data of these heating curves during the first 15 min and first 30 min, respectively. The temperature rise slopes of the four specimens (i.e., the bare copper panel, the carbon nanomaterial coating without dispersant, the carbon nanomaterial coating with dispersant, and the heat dissipation coating made of carbon nanomaterials + Al_2_O_3_) were compared. The corresponding heating slopes for the first 15 min were 5.41, 5.1, 5.09 and 4.98 °C/min and the slopes for the first 30 min were 3.1, 2.94, 2.89 and 2.68 °C/min, respectively. The linear regression analysis found that gradually adding carbon nanomaterials, dispersants and Al_2_O_3_ particles could reduce the temperature rise slope in order. In other words, they could gradually improve the heat-dissipating efficiency. The dispersant increased the dispersibility of the carbon nanomaterials in the coating, and therefore could indeed improve the heat-dissipating performance of the coating. The heat dissipation coating containing Al_2_O_3_ increased the heat conduction path so that the heat could transfer to the coating surface faster and then dissipate through heat radiation.

The infrared emissivities of the four different heat dissipation coatings (i.e., pure epoxy, epoxy + 30 wt% Al_2_O_3_, epoxy + 2 wt% GNFs + 2 wt% MWCNTs, epoxy + 30 wt% Al_2_O_3_ + 2 wt% GNFs + 2 wt% MWCNTs) were measured. Their corresponding emissivities were 0.93, 0.92, 0.98 and 0.96, respectively, as shown in Table 1. The surface of the pure epoxy had an emissivity of 0.93, which was reduced to 0.92 after adding 30 wt% Al_2_O_3_. When 4% carbon nanomaterials were added to the epoxy, the emissivity reached the highest value of 0.98, but this was slightly reduced to 0.96 after adding 30 wt% Al_2_O_3_. While the added Al_2_O_3_ particles slightly reduced the thermal emissivity, they helped the dispersion of the carbon nanomaterials during blending with the epoxy. Moreover, the Al_2_O_3_ particles also enabled the coating to obtain more heat conduction paths during the heat transfer process, thereby increasing the heat amount transferred to the surface and enhancing the heat-dissipating effect. Figure 9b shows the cooling performance of a 10 W heater, which reached a final equilibrium temperature of 128.2 °C for the coating that only contained carbon nanomaterials and reached 123.5 °C for the coating with carbon nanomaterials and Al_2_O_3_ particles. Compared with the final equilibrium temperature of 141.3 °C for the bare copper panel, the coating with carbon nanomaterials and Al_2_O_3_ particles achieved a temperature reduction of 17.8 °C.

Table 2 shows the comparison of the cooling performance of the heat dissipation coatings in the references [2,23,24,51]. These developed coatings used different matrix polymers, fillers made of carbon materials and inorganic particles, and surfactants used as dispersants to evaluate their heat-dissipating effect. The carbon materials filled into the coatings included graphite powders, carbon black powders, nanodiamond powders, carbon nanotubes and graphene flakes. The various surfactants were added into coatings as the dispersants of carbon materials to improve their cooling performance. These heat dissipation coatings made of carbon nanotubes and graphene flakes reached an emissivity of 0.98–0.99, which is better than that for carbon black and nanodiamond powders. In our study, a water-based epoxy was used as the polymer matrix to prepare the heat dissipation coatings. SDSS and PDDA surfactants were combined as a novel dispersant for dispersing carbon nanomaterials in aqueous suspensions. The infrared emissivity of the heat dissipation film was 0.96 under a filling of 30 wt% Al_2_O_3_ particles, 2 wt% GNFs and 2 wt% MWCNTs in the water-based epoxy. Such a heat dissipation film could reduce the thermal equilibrium temperature of the bare copper panel by 17.8 °C under a heating power of 10 W. This proves that the well-dispersed fillers have a synergistic effect in that they improve the heat-dissipating performance under the new type of carbon dispersant used; its performance was better than that of other heat dissipation coatings.

## 5. Conclusions

This study developed a heat dissipation coating that used GNFs, MWCNTs and Al_2_O_3_ particles as fillers blended into a water-based epoxy. Two novel surfactants, SDSS and PDDA, were also combined as a dispersant to improve the dispersibility of the carbon nanomaterials in the water-based epoxy. Based on the use of a 10 W heater as a heat source, the addition of the dispersant in the pure carbon nanomaterial (2 wt% GNFs + 2 wt% MWCNTs) coating could obtain a temperature difference of 13.1 °C, which was better by 4 °C than without dispersant. An additional 30 wt% Al_2_O_3_ particles were also added to the coating to further achieve a temperature difference of 17.8 °C, thus significantly promoting the heat-dissipating performance of the coating. The well-dispersed fillers produced a synergistic effect to improve the heat-dissipating performance of the coating. Under the optimal ratio of 30 wt% Al_2_O_3_ particles, 2 wt% GNFs and 2 wt% MWCNTs blended into a water-based epoxy, the infrared emissivity of the heat dissipation film was 0.96. The heat dissipation film could reduce the thermal equilibrium temperature of a bare copper panel to 17.8 °C under a heating power of 10 W. The application of the film in a cooling test using a 15 W LED bulb showed that its thermal equilibrium temperature could be reduced by 21.3 °C.

## Figures and Tables

**Table 1:** Infrared emissivity of various prepared coatings.

Types of coatings	IR Emissivity
Pure epoxy	0.93
Epoxy+30 wt% Al_2_O_3_	0.92
Epoxy+2 wt% GNFs+2 wt% MWCNTs	0.98
Epoxy+30 wt% Al_2_O_3_+2 wt% GNFs+2 wt% MWCNTs	0.96

**Table 2:** Comparison of the cooling performance and materials of various heat dissipation coatings in the references.

Matrix Polymer	Carbon Materials	Filler Particles	Added Surfactants	Infrared Emissivity	ΔT (°C)	Ref.
PTEE *^1^	Graphite powder	Silica	Triton X-100 *^2^ PEG *^3^	-	9	[22]
Acrylate	Nano diamond powder	-	FS-510 *^4^ PTFE *^5^	-	9	[2]
	Carbon black				11	
	MWCNTs				13	
Acrylic copolymer	Graphene	-	FS-510 PTFE SDBS *^6^	0.99	16	[23]
Acrylic copolymer	MWCNTs	-	SDBS	0.98	14	[24]
	Graphene nano-platelets	Alumina	-	0.9	11	[51]
Water-based epoxy	GNFs MWCNTs	Spherical alumina	SDSS *^7^ PDDA *^8^	0.96	17.8	**This study**

*^1^ PTEE: Thermoplastic polyester elastomer; *^2^ Triton X: Octylphenol ethoxylate polyoxyethylene 9.5-octylphenol; *^3^ PEG: Polyethylene glycol; *^4^ FS-510: Zonyl fluorosurfactant; *^5^ PTFE: Poly(tetraflouroethylene); *^6^ SDBS: Sodium dodecyl benzene sulfonate; *^7^ SDSS: Dihexyl sodium sulfosuccinate; *^8^ PDDA: Poly(dimethyldiallylammonium chloride).

## Data Availability

Not applicable.

## Supplementary Materials

The following are available online at https://www.mdpi.com/article/10.3390/polym14050952/s1, Figure S1: Schematic of the supercritical CO_2_ processing system for exfoliating graphene flakes. Figure S2: Graphene aqueous suspensions prepared using various surfactants. (a) Initial. (b) 1 hr. (c) 12 hr. (d) 3 days. Figure S3: CNT aqueous suspensions prepared using various surfactants. (a) Initial. (b) 3 min. (c) 10 min. (d) 3 days. Table S1: Linear regression analysis of heating curve.
