# Supplementary material for: Evaluation of Surfactants on Graphene Dispersion and Thermal Performance for Heat Dissipation Coating
Source: Polymers (Basel). 2022 Feb 27;14(5):952. doi: 10.3390/polym14050952 (PMC8912673; doi:10.3390/polym14050952)
Supplement: Supplementary file 1 [file polymers-14-00952-s001.zip › polymers-1567423-supplementary.pdf]

Supplementary Data

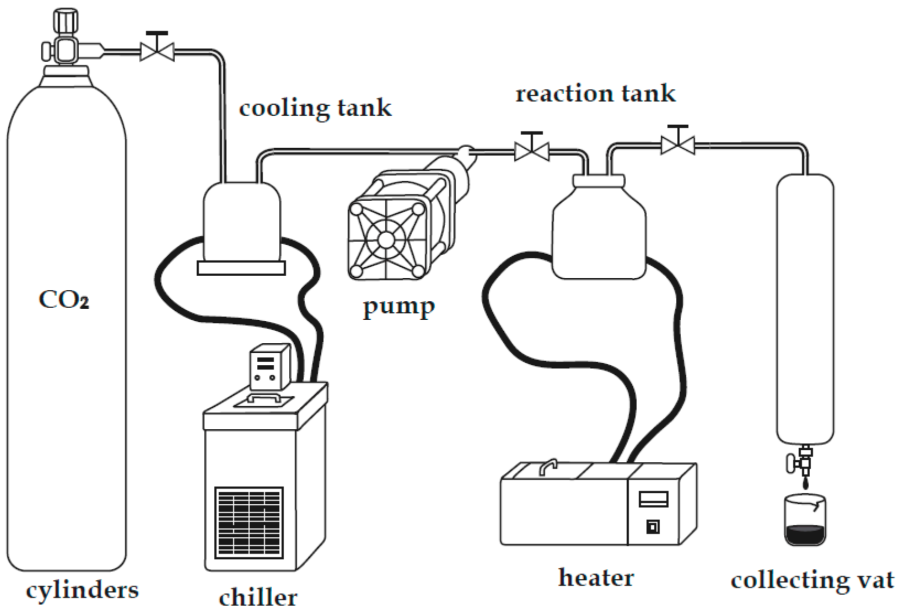

Figure S1. Schematic of the supercritical CO_2 processing system for exfoliating graphene flakes.

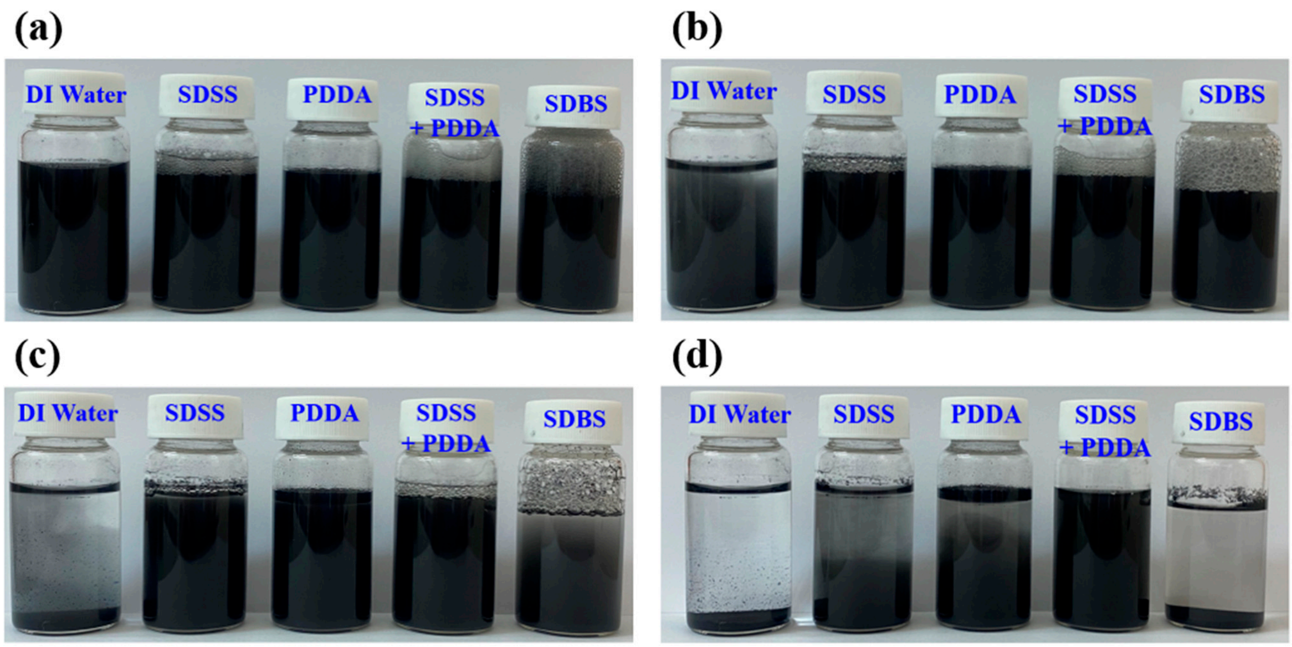

Figure S2. Graphene aqueous suspensions prepared using various surfactants. (a) Initial. (b) 1 hr. (c) 12 hr. (d) 3 days.

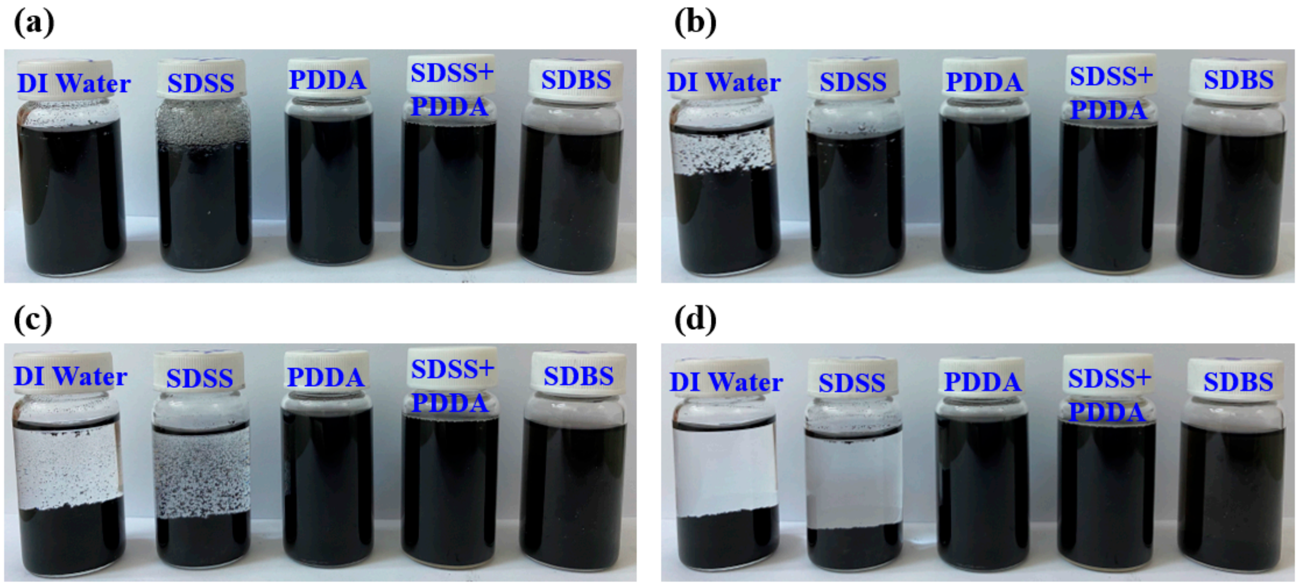

Figure S3. CNT aqueous suspensions prepared using various surfactants. (a) Initial. (b) 3 min. (c) 10 min. (d) 3 days.

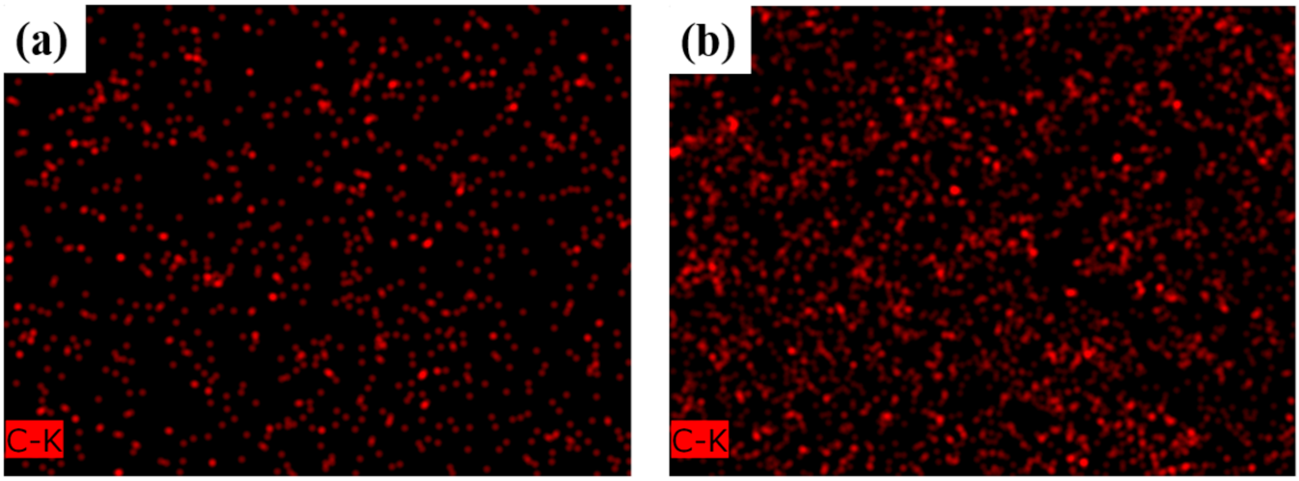

Figure S4. EDS images of the heat dissipation coatings: (a) with deionized water only, (b) with dispersant.

Table S1. Linear regression analysis of heating curve.

	Copper bare panel	4 wt% C materials + water	4 wt% C materials + dispersion	Al_2O_3 + 4 wt% C materials + dispersion
0-15 min	5.412	5.099	5.086	4.98
0-30 min	3.1	2.94	2.89	2.68
